# Global Aspects of Triazole Resistance in *Aspergillus fumigatus* with Focus on Latin American Countries

**DOI:** 10.3390/jof3010005

**Published:** 2017-02-10

**Authors:** Sarah Santos Gonçalves

**Affiliations:** Center for Research in Medical Mycology, Department of Pathology, Universidade Federal do Espírito Santo-UFES, Av. Marechal Campos, 1468, Maruípe CEP 29.040-090, Vitória-ES, Brazil; sarah.tavares@ufes.br or sarahunifesp@yahoo.com.br; Tel.: +55-27-3335-7290

**Keywords:** *Aspergillus fumigatus*, aspergillosis, Latin American, azole resistance, *CYP51A*

## Abstract

Azole resistance in *Aspergillus* has emerged as an escalating problem in health care, and it has been detected in patients exposed, or not, to these drugs. It is known that azole antifungals are widely applied not only in clinical treatments for fungal infections, but also as agricultural fungicides, resulting in a significant threat for human health. Although the number of cases of azole-resistant aspergillosis is still limited, various resistance mechanisms are described from clinical and environmental isolates. These mechanisms consist mainly of alterations in the target of azole action (*CYP51A* gene)—specifically on TR_34_/L98H and TR_46_/Y121F/T289A, which are responsible for over 90% of resistance cases. This review summarizes the epidemiology, management, and extension of azole resistance in *A. fumigatus* worldwide and its potential impact in Latin American countries, emphasizing its relevance to clinical practice.

## 1. An Overview of Aspergillosis in Contemporary Medicine

Members of the *Aspergillus* genus are ubiquitous saprobe fungi that can be found worldwide, occurring mainly in air, soil, water, plants, food, and inanimate surfaces [1]. *Aspergillus* is the most important pathogenic filamentous fungus in humans, causing a wide spectrum of clinical syndromes, highlighting invasive aspergillosis (IA), chronic pulmonary aspergillosis (CPA), and allergic bronchopulmonary aspergillosis (ABPA) [2,3,4].

In the invasive forms, the inhalation of *Aspergillus* conidia is the primary acquisition route, due to the high incidence of small airborne conidia. Therefore, the conidia can be inhaled and colonize the sinuses, afterwards evolving into the lower respiratory tract by specific circumstances [1,5]. Direct contact transmission has been associated with the contamination of biological prostheses and catheters. Moreover, the propagules can also be inoculated in the human host by trauma, causing cutaneous infections and fungal keratitis [1,6,7,8].

The substantial increase in the occurrence of IA is mainly due to a larger number of patients exposed to risk conditions, including primary immunodeficiency diseases, hematological malignancies, prolonged neutropenia or neutrophil disorders, corticosteroids (dose and duration), stem cell and solid organ transplantation, and a genetic disease called chronic granulomatous disease [9,10,11,12,13]. The mortality rates of IA are high and vary according to patient population, ranging from 38% in individuals with acute myeloid leukemia (AML), 50%–60% in solid organ transplant, and 70%–85% in other immunocompromised patients [11,14].

Unlike invasive aspergillosis, CPA occurs mostly in immunocompetent patients; it has been regarded as a major global health problem and is estimated to have a prevalence of 3 million cases [15,16]. This number is even higher when the issue is ABPA—it is estimated that this disease affects approximately 4 million adults [15]. It is worth mentioning that tuberculosis (particularly in developing countries), as well as the high occurrence of chronic obstructive pulmonary disease (COPD), contributes to the high rate of CPA, while the huge worldwide burden of asthma contributes to the incidence rate of ABPA [16,17,18].

In recent years, a large number of antifungal agents have become available for use in aspergillosis, such as echinocandins and new triazoles. On the other hand, different medical centers worldwide reported a large number of IA cases, with a treatment failure rate exceeding 50% of the cases evaluated [19,20]. Furthermore, individuals with CPA and ABPA represent the main patients with therapeutic failures due to triazole resistance, once they require long-term azole therapy [15,17].

There is a great interest in the detection of triazole-resistant strains in order to discriminate whether the determinants of treatment failure are related to the limitations of clinical and immunological conditions of the host, or to decreased antifungal activity associated with the drug used in the treatment [19,20]. In this context, this review summarizes the epidemiology, management, and extension of azole resistance in *A. fumigatus* worldwide and its potential impact in Latin American countries, emphasizing its relevance to clinical practice.

## 2. Methods

Articles regarding triazole resistance in *A. fumigatus* were identified and reviewed using the Scientific Electronic Library Online and Medline databases through January 2017. Articles were reviewed regardless of the language or the date of publication, and were retrieved using the following keywords: *Aspergillus fumigatus*, aspergillosis, antifungal susceptibility, azole resistance, *CYP51A*. Each term was combined with the following keywords: Latin America, South America, developing world, Central America, Colombia, and Brazil. During the analysis, an exhaustive effort was made to collect all available information on epidemiology, clinical implications, and management of triazole resistance worldwide and in Latin America.

## 3. Global Scenario of Resistance to Azoles

In antifungal resistance involving *Aspergillus*, two events have been observed: (i) primary or intrinsic resistance to azole and other antifungal agents; and (ii) secondary or acquired azole resistance in *A. fumigatus* isolates [21].

Primary resistance to amphotericin B is well recognized in *A. terreus*, and in some *A. flavus* and *A. ustus* isolates [21,22,23,24]. Different species of *Aspergillus* genus belonging to the same section may present distinct antifungal susceptibility profiles. Recently, it has also been demonstrated in several species in the section *Fumigati*. It is known, for example, that *A. lentulus*, *A. udagawae*, *A. pseudofisheri*, *A. fumigatiffinis*, *A. thermomutatus*, and *A. viridinutans (Aspergillus* section *Fumigati*) have shown decreased susceptibility to several antifungal agents, including amphotericin B, azoles, and echinocandins [19,25,26,27].

More recently, a multicenter international surveillance network reported a rate of triazole resistance of 3.2% amidst section *Fumigati* strains. Among all resistant isolates, 78% were *A. fumigatus sensu stricto*, and 22% were sibling species (*A. udagawae*, *A. thermomutatus*, and *A. lentulus*) [2,28]. In 2015, Bastos et al. [29] described the first Brazilian case of pulmonary invasive aspergillosis caused by *A. lentulus*, reporting a strain resistant to amphotericin B and azoles (itraconazole and voriconazole). In another Brazilian study, high minimum inhibitory concentration (MIC) values against itraconazole and voriconazole (2 and 16 µg/mL, respectively) were observed for one isolate of *A. thermomutatus* [30]. Considering that different species may present distinct susceptibility profiles, species identification has become an important predictor of the clinical outcome [31].

Although *A. fumigatus* is generally susceptible to these azole antifungals, acquired resistance is increasingly being reported over the last few years [32,33,34]. These antifungal agents have affinity for heme prosthetic group, found in several enzymes participating in ergosterol biosynthesis—an essential component of the fungal cell membrane [35]. The specific target of azoles is the cytochrome P450-dependent enzyme lanosterol 14*α*-demethylase (Cyp51A). The main resistance mechanism appears to be mutations in genes (*CYP51A* and *CYP51B*) encoding the azole target enzyme, which accounts for over 90% of resistance cases [36,37].

Some mutations are responsible for multi-azole resistance, and such resistance has a great impact on the outcome of patients with both invasive aspergillosis and chronic disease [38,39]. Most of the azole resistant strains harbor the TR_34_/L98H mutation in the *CYP51A* gene, which consists of substitutions of leucine 98 for histidine (L98H) in addition to the presence of two copies of a 34-bp sequence tandem in the promoter of the *CYP51A* gene, resulting in an overexpression of the *CYP51A* [34,35,40,41,42,43]. These mutations have been described as occurring in environmental and clinical isolates of *A. fumigatus* found in numerous countries in the Europe, China, the Middle East, India, Africa, Australia, Turkey, and most recently in Colombia [17,44].

Recently, a novel *CYP51A*-mediated resistance mutation that leads to high-level voriconazole resistance—TR_46_/Y121F/T289A—has been also described as occurring in environmental and clinical isolates of *A. fumigatus* found in Europe, India, Africa, and Latin America [17,35,44,45,46,47,48,49,50,51,52,53,54].

Specific mutations in *CYP51A* may confer resistance to one, two, or all triazoles. In fact, these mutations have not only been associated with resistance to itraconazole, posaconazole, and voriconazole, but also with other azoles. Isavuconazole—a triazole which has only recently been licensed for use in humans—showed decreased in vitro activity and reduced in vivo efficacy against *A. fumigatus* strains harboring the resistance mechanisms described above [17,55,56,57,58].

It is interesting to comment that there has been a predominance of TR_34_/L98H and TR_46_/Y121F/T289A resistance mechanisms in clinical isolates from patients exposed, or not, to these drugs, as well as in environmental isolates. Between 64% and 71% of multi-azole resistance in *A. fumigatus* strains has been reported in patients with IA who were not previously exposed to azole treatments and who could be considered azole-naive [4,41,59,60].

Concurrent genetic studies of worldwide *A. fumigatus* isolates harboring the TR_34_/L98H resistance mechanism suggested clonal expansion of a common resistant ancestor [35,61]. This could easily occur, given that environmental dissemination of conidia can be facilitated due to their tiny size and their ability to cover thousands of miles in the air [1,6,17,62,63]. In addition, *Aspergillus* spores are released from different reservoirs, and often remain in the air for prolonged periods [1,6].

Another route of azole resistance selection is through long-term azole exposure, particularly common in individuals with chronic forms of aspergillosis (aspergillomas: high fungal burden and chronic cavitary pulmonary) [17,19,64]. In this case, some researchers suggest that there is evidence of fungal evolution within the lung, since strains of identical genotype with distinct susceptibility profiles have been detected [65]. Azole resistance has been common in this population of patients, and the resistance mechanisms are quite varied and—at first—caused by non-environmental mutations [17,64]. Moreover, multiple resistance mechanisms can be found in different colonies from a single specimen [17].

Currently, azole resistance has been much debated, given that it is an aggravating threat to human health, since the management of patients with resistant isolates and its detection is extremely complex, and there are not established guidelines. Furthermore, is important to highlight that these mutations have been found worldwide, signaling a possible extension of the problem [4].

## 4. Epidemiology of Triazole Resistance in *A. fumigatus* in Latin American Countries

**Figure 1:**
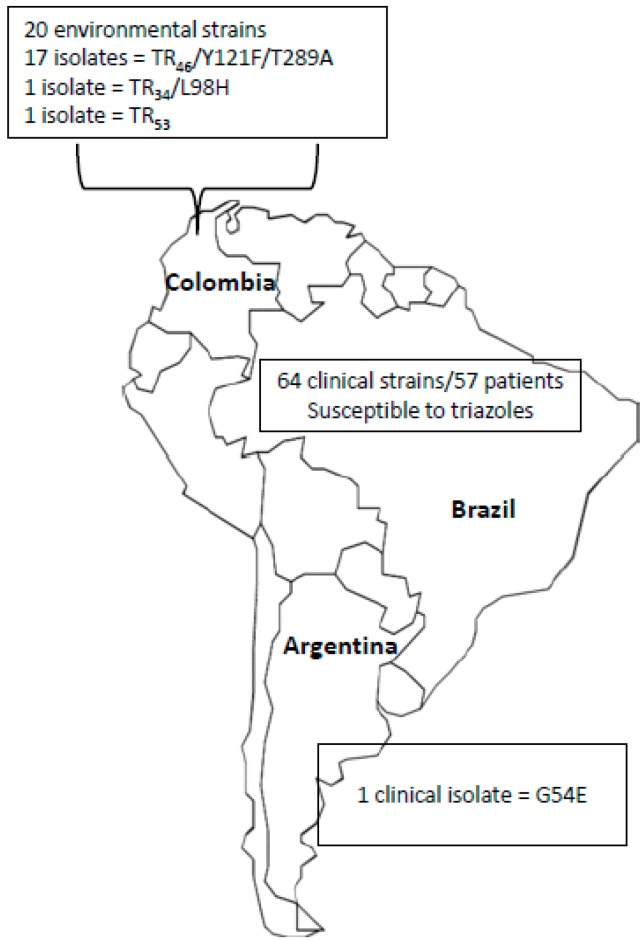
Epidemiology of azole resistance in Latin American Countries and the main resistance mechanisms.

Although the triazole resistance in *A. fumigatus* has been an extensively debated topic, little is known about this problem in Latin America. Until now, only three studies evaluated strains recovered from these regions, as illustrated in Figure 1.

Le Pape et al. [44] evaluated 60 soil samples from flower fields and greenhouses in the outskirts of Bogotá, Colombia. The isolates were recovered from a region with a high usage of tebuconazole and difeconazole. From these samples, 20 *A. fumigatus* strains were evaluated for *CYP51A* gene alterations. Among the strains studied, 19 showed changes in *CYP51A* gene, with 17 isolates presenting TR_46_/Y121F/T289A, 1 with TR_34_/L98H and 1 with TR_53_. In this study, no clinical isolates were investigated.

Van der Linden et al. [28] performed a multicenter international surveillance network in order to determine the prevalence of azole resistance in clinical *Aspergillus* isolates in several countries worldwide. A total of 3788 *Aspergillus* isolates were screened in 22 medical centers from 19 countries. Among these isolates, 64 strains were recovered from 57 Brazilian patients. Triazole-resistant phenotypes were not detected in Brazilian isolates by phenotypic screening-method using a four-well plate format with agar supplemented with itraconazole. Unlike the Colombian study, only clinical isolates were evaluated. In addition, the amount of strains investigated was not representative of the whole country, given that the strains were collected in two out of 26 federated states. On the other hand, it is worth noting that the presence of gene changes does not necessarily indicate in vitro resistance.

More recently, Leonardelli et al. [8] described the first report in South America of a clinical *A. fumigatus* strain carrying the substitution G54E at *CYP51A* associated with itraconazole resistance from an Argentine patient with fungal keratitis. Considering that the patient had never received any treatment with azole before, the authors suggested that the patient may have acquired this resistant isolate from the environment.

On the basis of these studies, azole resistance in Latin America is an issue that needs to be extensively explored, because it displays the two relevant scenarios for the development of triazole resistance in *A. fumigatus*: (i) environmental route due to increased usage of pesticides in agriculture; and (ii) prolonged azole exposure in CPA patients, although this disease is neglected in Latin America, particularly in geographic areas where tuberculosis is endemic. In the latter case, it is worth mentioning that there is a large contingent of patients with tuberculosis that evolves with residual cavities at the end of treatment of mycobacteriosis, becoming patients at risk for the development of CPA throughout life.

Therefore, there is a need to conduct antifungal resistance surveillance studies using clinical and environmental isolates from different geographic regions in patients exposed, or not, to these drugs, in order to establish the adequate management of patient and infection control.

## 5. Clinical Implications and the Management of Azole-Resistant Aspergillosis

Currently, various antifungal drugs for systemic use are commercially available for the treatment of invasive fungal infections, including different amphotericin B formulations, 5-fluorocytosine, triazoles (itraconazole, voriconazole, posaconazole, and isavuconazole), and echinocandin [19,20]. According to the latest guidelines elaborated by Walsh et al. [66], voriconazole is the first drug of choice for the treatment of IA, and lipid formulations of amphotericin B (L-AmB) are an effective alternative. Moreover, posaconazole and caspofungin are recommended in the treatment of refractory IA or in patients intolerant to other therapies. Azole antifungals are still recommended for aspergillosis prophylaxis in hematopoietic stem cell transplantation (HSCT) recipients with graft-versus-host disease (GVHD) and in neutropenic patients with acute myelogenous leukemia or myelodysplastic syndrome [66]. However, as discussed earlier, the use of triazoles has been compromised by the emergence of azole resistance in *A. fumigatus* [17].

Various reports indicated a high mortality rate in patients with documented azole-resistant IA [41,67]. Given the high and rising triazole resistance rates and the absence of management guidelines for patients with aspergillosis, a group that comprised 21 experts (representatives of medical, microbiological and pharmacological areas) from 11 countries proposed some changes in the treatment of patients with azole-resistant *A. fumigatus* [17]. Briefly, some recommendations are described below.

For patients with IA in regions with epidemiological evidence of azole resistance in environmental isolates, new therapeutic strategies were suggested. Recommendations were elaborated based two parameters: (i) levels of environmental azole resistance of 5% to 10%; and (ii) azole resistance rate >10%.

In the first case (resistance rate due to environmental mechanism of 5% to 10%), approximately half the panel advocated for voriconazole as the first-line therapy, while the other half backed the usage of l-AmB or a combination of voriconazole and an echinocandin. On the other hand, in regions with an environmental azole resistance rate >10%, the panel advocated either for a combination of voriconazole plus an echinocandin or l-AmB monotherapy as initial empiric therapy. In fact, these parameters were recommended in IA with these levels of resistance, pending susceptibility data.

In cases of CPA, the management of patients is even more complicated, assuming that these individuals require long-term oral treatment, and azoles are the only oral agent available. Therefore, the panel suggested that these drugs should remain as the first line therapy regardless of environmental resistance rates. During the course of the disease, if a patient develops resistance to one or two azoles, it was agreed that therapy should be replaced by an alternative azole to which the fungus is susceptible. Intravenous therapy with a non-azole agent was highly advised in the case of pan-azole resistance. It is important to mention the same management principles were also considered for patients with ABPA, *Aspergillus* bronchitis, and severe asthma with fungal sensitization.

Early diagnosis of resistance—mainly in regions with high azole resistance rates—is another factor that may contribute to the adequate management of patients. The same expert group mentioned above agreed that clinical samples should be recovered prior to therapy, and tested using culture-based methods for patients with IA. In this case, all *Aspergillus* isolates obtained should be identified to species section level and have their susceptibility profile established by reference laboratories or the local clinical laboratory [68]. If an azole-resistant *A. fumigatus* is identified, molecular resistance mechanisms should be detected for epidemiological purposes [67].

Accordingly, in order to improve the diagnosis in patients with CPA, some recommendations were proposed by a panel of experts, highlighting: (i) regular culture and susceptibility testing of all isolates from patients on long-term azole therapy; and (ii) testing of multiples colonies from sputum cultures and using primary plates to detect resistance [69].

Unluckily, microbiological diagnosis of aspergillosis and triazole resistance is limited by poor culture yield [3,70]. Considering the low efficiency of culture, commercial methods that allow fast and accurate detection of azole resistance need to be validated from primary samples [3,67,70]. When this is not possible, susceptibility testing of *A. fumigatus* isolates prior to and during antifungal treatment can be a useful tool instead.

In resource-scarce countries (as is the case of most Latin American countries), clinical implications regarding early diagnosis and effective treatment of azole-resistant aspergillosis are limiting factors in patient management, representing a huge challenge. Additionally, alternative therapeutic options such as l-AmB have the highest cost and require adequate medical infrastructures to allow intravenous administration [4].

## 6. Final Considerations

Although azole resistance has been reported in six continents, little is known about the actual frequency of azole resistance in *Aspergillus* isolates globally, mainly because most medical centers do not perform routine susceptibility testing. Moreover, azole resistance may be underestimated by culture-based diagnosis [3,67,70]. Diagnosis of azole resistance in negative cultures is a substantial challenge, since the current biomarkers—such as galactomannan and 1.3 β, d-glucan—are not permissible for identification of species and still less for performing in vitro susceptibility testing.

Information about azole resistance is still very limited in Latin American countries. Although only two studies detected azole resistance in environmental and clinical isolates, this does not mean that they are exempt from this problem—it means that they are unaware of it. However, it is essential to join efforts to promote monitoring of clinical and environmental isolates as a key to a better understanding of the magnitude of the problem.

## References

[1] Nicolle M.C., Benet T., Vanhems P. (2011). Aspergillosis: Nosocomial or community-acquired?. Med. Mycol..

[2] Lamoth F. (2016). *Aspergillus fumigatus*-related species in clinical practice. Front. Microbiol..

[3] Zhao Y., Garnaud C., Brenier-Pinchart M.P., Thiebaut-Bertrand A., Saint-Raymond C., Camara B., Hamidfar R., Cognet O., Maubon D., Cornet M. (2016). Direct molecular diagnosis of aspergillosis and *CYP51A* profiling from respiratory samples of french patients. Front. Microbiol..

[4] Chang H., Ashu E., Sharma C., Kathuria S., Chowdhary A., Xu J. (2016). Diversity and origins of indian multi-triazole resistant strains of *Aspergillus fumigatus*. Mycoses.

[5] Pasqualotto A.C. (2009). Differences in pathogenicity and clinical syndromes due to *Aspergillus fumigatus* and *Aspergillus flavus*. Med. Mycol..

[6] Warris A., Voss A., Verweij P.E. (2001). Hospital sources of aspergillus: New routes of transmission?. Rev. Iberoam. Micol..

[7] Pfaller M.A., Diekema D.J. (2010). Epidemiology of invasive mycoses in North America. Crit. Rev. Microbiol..

[8] Leonardelli F., Theill L., Nardin M.E., Macedo D., Dudiuk C., Mendez E., Gamarra S., Garcia-Effron G. (2017). First itraconazole resistant *Aspergillus fumigatus* clinical isolate harbouring a G54E substitution in *CYP51AP* in South America. Rev. Iberoam. Micol..

[9] Nucci M., Queiroz-Telles F., Tobon A.M., Restrepo A., Colombo A.L. (2010). Epidemiology of opportunistic fungal infections in Latin America. Clin. Infect. Dis..

[10] Stevens D.A. (2009). Clinical aspergillosis for basic scientists. Med. Mycol..

[11] Pagano L., Caira M., Candoni A., Offidani M., Martino B., Specchia G., Pastore D., Stanzani M., Cattaneo C., Fanci R. (2010). Invasive aspergillosis in patients with acute myeloid leukemia: A seifem-2008 registry study. Haematologica.

[12] Lanternier F., Cypowyj S., Picard C., Bustamante J., Lortholary O., Casanova J.L., Puel A. (2013). Primary immunodeficiencies underlying fungal infections. Curr. Opin. Pediatr..

[13] Antachopoulos C., Walsh T.J., Roilides E. (2007). Fungal infections in primary immunodeficiencies. Eur. J. Pediatr..

[14] Lass-Florl C., Griff K., Mayr A., Petzer A., Gastl G., Bonatti H., Freund M., Kropshofer G., Dierich M.P., Nachbaur D. (2005). Epidemiology and outcome of infections due to *Aspergillus terreus*: 10-year single centre experience. Br. J. Haematol..

[15] Denning D.W., Perlin D.S. (2011). Azole resistance in aspergillus: A growing public health menace. Future Microbiol..

[16] Denning D.W., Pleuvry A., Cole D.C. (2011). Global burden of chronic pulmonary aspergillosis as a sequel to pulmonary tuberculosis. Bull. World Health Organ..

[17] Verweij P.E., Ananda-Rajah M., Andes D., Arendrup M.C., Bruggemann R.J., Chowdhary A., Cornely O.A., Denning D.W., Groll A.H., Izumikawa K. (2015). International expert opinion on the management of infection caused by azole-resistant *Aspergillus fumigatus*. Drug Resist. Updat..

[18] Brown G.D., Denning D.W., Gow N.A., Levitz S.M., Netea M.G., White T.C. (2012). Hidden killers: Human fungal infections. Sci. Transl. Med..

[19] Howard S.J., Arendrup M.C. (2011). Acquired antifungal drug resistance in *Aspergillus fumigatus*: Epidemiology and detection. Med. Mycol..

[20] Shi J.Y., Xu Y.C., Shi Y., Lu H.X., Liu Y., Zhao W.S., Chen D.M., Xi L.Y., Zhou X., Wang H. (2010). In vitro susceptibility testing of aspergillus spp. Against voriconazole, itraconazole, posaconazole, amphotericin b and caspofungin. Chin. Med. J..

[21] Goncalves S.S., Souza A.C., Chowdhary A., Meis J.F., Colombo A.L. (2016). Epidemiology and molecular mechanisms of antifungal resistance in Candida and Aspergillus. Mycoses.

[22] Goncalves S.S., Stchigel A.M., Cano J., Guarro J., Colombo A.L. (2013). In vitro antifungal susceptibility of clinically relevant species belonging to aspergillus section flavi. Antimicrob. Agents Chemother..

[23] Koss T., Bagheri B., Zeana C., Romagnoli M.F., Grossman M.E. (2002). Amphotericin B-resistant *Aspergillus flavus* infection successfully treated with caspofungin, a novel antifungal agent. J. Am. Acad. Dermatol..

[24] Azzola A., Passweg J.R., Habicht J.M., Bubendorf L., Tamm M., Gratwohl A., Eich G. (2004). Use of lung resection and voriconazole for successful treatment of invasive pulmonary aspergillus ustus infection. J. Clin. Microbiol..

[25] Baddley J.W., Marr K.A., Andes D.R., Walsh T.J., Kauffman C.A., Kontoyiannis D.P., Ito J.I., Balajee S.A., Pappas P.G., Moser S.A. (2009). Patterns of susceptibility of aspergillus isolates recovered from patients enrolled in the transplant-associated infection surveillance network. J. Clin. Microbiol..

[26] Balajee S.A., Kano R., Baddley J.W., Moser S.A., Marr K.A., Alexander B.D., Andes D., Kontoyiannis D.P., Perrone G., Peterson S. (2009). Molecular identification of Aspergillus species collected for the transplant-associated infection surveillance network. J. Clin. Microbiol..

[27] Krishnan S., Manavathu E.K., Chandrasekar P.H. (2009). Aspergillus flavus: An emerging non-fumigatus aspergillus species of significance. Mycoses.

[28] Van der Linden J.W., Arendrup M.C., Warris A., Lagrou K., Pelloux H., Hauser P.M., Chryssanthou E., Mellado E., Kidd S.E., Tortorano A.M. (2015). Prospective multicenter international surveillance of azole resistance in *Aspergillus fumigatus*. Emerg. Infect. Dis..

[29] Bastos V.R., Santos D.W., Padovan A.C., Melo A.S., Mazzolin Mde A., Camargo L.F., Colombo A.L. (2015). Early invasive pulmonary aspergillosis in a kidney transplant recipient caused by *Aspergillus lentulus*: First brazilian report. Mycopathologia.

[30] Negri C.E., Goncalves S.S., Xafranski H., Bergamasco M.D., Aquino V.R., Castro P.T., Colombo A.L. (2014). Cryptic and rare *Aspergillus* species in Brazil: Prevalence in clinical samples and in vitro susceptibility to triazoles. J. Clin. Microbiol..

[31] Nascimento A.M., Goldman G.H., Park S., Marras S.A., Delmas G., Oza U., Lolans K., Dudley M.N., Mann P.A., Perlin D.S. (2003). Multiple resistance mechanisms among *Aspergillus fumigatus* mutants with high-level resistance to itraconazole. Antimicrob. Agents Chemother..

[32] Camps S.M., van der Linden J.W., Li Y., Kuijper E.J., van Dissel J.T., Verweij P.E., Melchers W.J. (2012). Rapid induction of multiple resistance mechanisms in *Aspergillus fumigatus* during azole therapy: A case study and review of the literature. Antimicrob. Agents Chemother..

[33] Verweij P.E., Snelders E., Kema G.H., Mellado E., Melchers W.J. (2009). Azole resistance in *Aspergillus fumigatus*: A side-effect of environmental fungicide use?. Lancet Infect. Dis..

[34] Snelders E., van der Lee H.A., Kuijpers J., Rijs A.J., Varga J., Samson R.A., Mellado E., Donders A.R., Melchers W.J., Verweij P.E. (2008). Emergence of azole resistance in *Aspergillus fumigatus* and spread of a single resistance mechanism. PLoS Med..

[35] Abdolrasouli A., Rhodes J., Beale M.A., Hagen F., Rogers T.R., Chowdhary A., Meis J.F., Armstrong-James D., Fisher M.C. (2015). Genomic context of azole resistance mutations in *Aspergillus fumigatus* determined using whole-genome sequencing. MBio.

[36] Mellado E., Diaz-Guerra T.M., Cuenca-Estrella M., Rodriguez-Tudela J.L. (2001). Identification of two different 14-α sterol demethylase-related genes (*CYP51A* and *CYP51B*) in *Aspergillus fumigatus* and other aspergillus species. J. Clin. Microbiol..

[37] Moye-Rowley W.S. (2015). Multiple mechanisms contribute to the development of clinically significant azole resistance in *Aspergillus fumigatus*. Front. Microbiol..

[38] Lazzarini C., Esposto M.C., Prigitano A., Cogliati M., De Lorenzis G., Tortorano A.M. (2015). Azole resistance in *Aspergillus fumigatus* clinical isolates from an italian culture collection. Antimicrob. Agents Chemother..

[39] Denning D.W., Bowyer P. (2013). Voriconazole resistance in *Aspergillus fumigatus*: Should we be concerned?. Clin. Infect. Dis..

[40] Mellado E., Garcia-Effron G., Alcazar-Fuoli L., Melchers W.J., Verweij P.E., Cuenca-Estrella M., Rodriguez-Tudela J.L. (2007). A new *Aspergillus fumigatus* resistance mechanism conferring in vitro cross-resistance to azole antifungals involves a combination of *CYP51A* alterations. Antimicrob. Agents Chemother..

[41] Van der Linden J.W., Snelders E., Kampinga G.A., Rijnders B.J., Mattsson E., Debets-Ossenkopp Y.J., Kuijper E.J., Van Tiel F.H., Melchers W.J., Verweij P.E. (2011). Clinical implications of azole resistance in *Aspergillus fumigatus*, the netherlands, 2007–2009. Emerg. Infect. Dis..

[42] Shapiro R.S., Robbins N., Cowen L.E. (2011). Regulatory circuitry governing fungal development, drug resistance, and disease. Microbiol. Mol. Biol. Rev..

[43] Van Ingen J., van der Lee H.A., Rijs T.A., Zoll J., Leenstra T., Melchers W.J., Verweij P.E. (2015). Azole, polyene and echinocandin mic distributions for wild-type, TR34/L98H AND TR46/Y121F/T289A *Aspergillus fumigatus* isolates in The Netherlands. J. Antimicrob. Chemother..

[44] Le Pape P., Lavergne R.A., Morio F., Alvarez-Moreno C. (2016). Multiple fungicide-driven alterations in azole-resistant *Aspergillus fumigatus*, colombia, 2015. Emerg. Infect. Dis..

[45] Fuhren J., Voskuil W.S., Boel C.H., Haas P.J., Hagen F., Meis J.F., Kusters J.G. (2015). High prevalence of azole resistance in *Aspergillus fumigatus* isolates from high-risk patients. J. Antimicrob. Chemother..

[46] Tashiro M., Izumikawa K., Hirano K., Ide S., Mihara T., Hosogaya N., Takazono T., Morinaga Y., Nakamura S., Kurihara S. (2012). Correlation between triazole treatment history and susceptibility in clinically isolated *Aspergillus fumigatus*. Antimicrob. Agents Chemother..

[47] Fischer J., van Koningsbruggen-Rietschel S., Rietschel E., Vehreschild M.J., Wisplinghoff H., Kronke M., Hamprecht A. (2014). Prevalence and molecular characterization of azole resistance in *Aspergillus* spp. Isolates from german cystic fibrosis patients. J. Antimicrob. Chemother..

[48] Astvad K.M., Jensen R.H., Hassan T.M., Mathiasen E.G., Thomsen G.M., Pedersen U.G., Christensen M., Hilberg O., Arendrup M.C. (2014). First detection of TR46/Y121F/T289A and TR34/L98H alterations in *Aspergillus fumigatus* isolates from azole-naive patients in Denmark despite negative findings in the environment. Antimicrob. Agents Chemother..

[49] Chowdhary A., Sharma C., Kathuria S., Hagen F., Meis J.F. (2014). Azole-resistant *Aspergillus fumigatus* with the environmental TR46/Y121F/T289A mutation in India. J. Antimicrob. Chemother..

[50] Chowdhary A., Sharma C., Hagen F., Meis J.F. (2014). Exploring azole antifungal drug resistance in *Aspergillus fumigatus* with special reference to resistance mechanisms. Future Microbiol..

[51] Chen Y., Wang H., Lu Z., Li P., Zhang Q., Jia T., Zhao J., Tian S., Han X., Chen F. (2015). Emergence of TR46/Y121F/T289A in an *Aspergillus fumigatus* isolate from a Chinese patient. Antimicrob. Agents Chemother..

[52] Pelaez T., Monteiro M.C., Garcia-Rubio R., Bouza E., Gomez-Lopez A., Mellado E. (2015). First detection of *Aspergillus fumigatus* azole-resistant strain due to *CYP51A* TR46/Y121F/T289A in an azole-naive patient in spain. New Microbes New Infect..

[53] Steinmann J., Hamprecht A., Vehreschild M.J., Cornely O.A., Buchheidt D., Spiess B., Koldehoff M., Buer J., Meis J.F., Rath P.M. (2015). Emergence of azole-resistant invasive aspergillosis in hsct recipients in germany. J. Antimicrob. Chemother..

[54] Lavergne R.A., Morio F., Favennec L., Dominique S., Meis J.F., Gargala G., Verweij P.E., Le Pape P. (2015). First description of azole-resistant *Aspergillus fumigatus* due to TR46/Y121F/T289A mutation in france. Antimicrob. Agents Chemother..

[55] Howard S.J., Pasqualotto A.C., Anderson M.J., Leatherbarrow H., Albarrag A.M., Harrison E., Gregson L., Bowyer P., Denning D.W. (2013). Major variations in *Aspergillus fumigatus* arising within aspergillomas in chronic pulmonary aspergillosis. Mycoses.

[56] Gregson L., Goodwin J., Johnson A., McEntee L., Moore C.B., Richardson M., Hope W.W., Howard S.J. (2013). In vitro susceptibility of *Aspergillus fumigatus* to isavuconazole: Correlation with itraconazole, voriconazole, and posaconazole. Antimicrob. Agents Chemother..

[57] Lepak A.J., Marchillo K., Vanhecker J., Andes D.R. (2013). Isavuconazole (BAL4815) pharmacodynamic target determination in an in vivo murine model of invasive pulmonary aspergillosis against wild-type and CYP51 mutant isolates of *Aspergillus fumigatus*. Antimicrob. Agents Chemother..

[58] Seyedmousavi S., Bruggemann R.J., Meis J.F., Melchers W.J., Verweij P.E., Mouton J.W. (2015). Pharmacodynamics of isavuconazole in an *Aspergillus fumigatus* mouse infection model. Antimicrob. Agents Chemother..

[59] Sanglard D. (2016). Emerging threats in antifungal-resistant fungal pathogens. Front Med.

[60] Van der Linden J.W., Camps S.M., Kampinga G.A., Arends J.P., Debets-Ossenkopp Y.J., Haas P.J., Rijnders B.J., Kuijper E.J., van Tiel F.H., Varga J. (2013). Aspergillosis due to voriconazole highly resistant *Aspergillus fumigatus* and recovery of genetically related resistant isolates from domiciles. Clin. Infect. Dis..

[61] Jensen R.H., Hagen F., Astvad K.M., Tyron A., Meis J.F., Arendrup M.C. (2016). Azole-resistant *Aspergillus fumigatus* in Denmark: A laboratory-based study on resistance mechanisms and genotypes. Clin. Microbiol. Infect..

[62] Vermeulen E., Lagrou K., Verweij P.E. (2013). Azole resistance in *Aspergillus fumigatus*: A growing public health concern. Curr. Opin. Infect. Dis..

[63] Chowdhary A., Kathuria S., Xu J., Meis J.F. (2013). Emergence of azole-resistant *Aspergillus fumigatus* strains due to agricultural azole use creates an increasing threat to human health. PLoS Pathog..

[64] Howard S.J., Cerar D., Anderson M.J., Albarrag A., Fisher M.C., Pasqualotto A.C., Laverdiere M., Arendrup M.C., Perlin D.S., Denning D.W. (2009). Frequency and evolution of azole resistance in *Aspergillus fumigatus* associated with treatment failure. Emerg. Infect. Dis..

[65] Mortensen K.L., Jensen R.H., Johansen H.K., Skov M., Pressler T., Howard S.J., Leatherbarrow H., Mellado E., Arendrup M.C. (2011). Aspergillus species and other molds in respiratory samples from patients with cystic fibrosis: A laboratory-based study with focus on *Aspergillus fumigatus* azole resistance. J. Clin. Microbiol..

[66] Walsh T.J., Anaissie E.J., Denning D.W., Herbrecht R., Kontoyiannis D.P., Marr K.A., Morrison V.A., Segal B.H., Steinbach W.J., Stevens D.A. (2008). Treatment of aspergillosis: Clinical practice guidelines of the infectious diseases society of America. Clin. Infect. Dis..

[67] Verweij P.E., Chowdhary A., Melchers W.J., Meis J.F. (2016). Azole resistance in *Aspergillus fumigatus*: Can we retain the clinical use of mold-active antifungal azoles?. Clin. Infect. Dis..

[68] Schelenz S., Barnes R.A., Barton R.C., Cleverley J.R., Lucas S.B., Kibbler C.C., Denning D.W., on behalf of the British Society for Medical Mycology (2015). British society for medical mycology best practice recommendations for the diagnosis of serious fungal diseases. Lancet Infect. Dis..

[69] Fraczek M.G., Kirwan M.B., Moore C.B., Morris J., Denning D.W., Richardson M.D. (2014). Volume dependency for culture of fungi from respiratory secretions and increased sensitivity of aspergillus quantitative pcr. Mycoses.

[70] Van der Linden J.W., Arendrup M.C., Melchers W.J., Verweij P.E. (2016). Azole resistance of *Aspergillus fumigatus* in immunocompromised patients with invasive aspergillosis. Emerg. Infect. Dis..

